# Supplementary material for: Low mammalian species richness is associated with Kyasanur Forest disease outbreak risk in deforested landscapes in the Western Ghats, India
Source: One Health. 2021 Aug 5;13:100299. doi: 10.1016/j.onehlt.2021.100299 (PMC8367838; doi:10.1016/j.onehlt.2021.100299)

S1 Table 1. Species distribution ensemble models based on the weighted means of boosted regression tree-, random forest-, and generalised additive model-based species distributions. Performance is indicated by the area under the receiver operating characteristic curve (AUC) and fit is indicated by the deviance. The true skill statistic was used to mark the cutoff of the estimated species distribution function demarcating species presence.

**Mammal species**	**Observations**	**AUC (%)**	**TSS**	**Deviance**
Axis axis	185	93	0.80	0.57
Macaca radiata	359	88	0.69	0.88
Funambulus palmarum	239	90	0.69	0.77
Ratufa indica	217	95	0.88	0.49
Bos gaurus	153	92	0.76	0.69
Elephas maximus	145	96	0.85	0.47
Pteropus giganteus	138	94	0.81	0.61
Panthera tigris	106	95	0.81	0.52
Sus scrofa	108	88	0.69	0.75
Rusa unicolor	97	97	0.82	0.50
Gazella bennettii	88	100	0.98	0.36
Semnopithecus hypoleucos	92	86	0.66	0.93
Semnopithecus priam	56	87	0.65	0.87
Funambulus tristriatus	50	96	0.90	0.51
Semnopithecus johnii	50	91	0.87	0.65
Semnopithecus dussumieri	48	87	0.64	0.87
Funambulus pennantii	43	89	0.71	0.78
Antilope cervicapra	42	83	0.60	0.91
Canis aureus	52	85	0.66	0.92
Cuon alpinus	40	95	0.90	0.50
Macaca silenus	34	81	0.61	0.87
Panthera pardus	34	77	0.50	0.99
Melursus ursinus	33	90	0.78	0.67
Lepus nigricollis	32	84	0.67	0.88

S2 Figure 1. Individual species distributions.

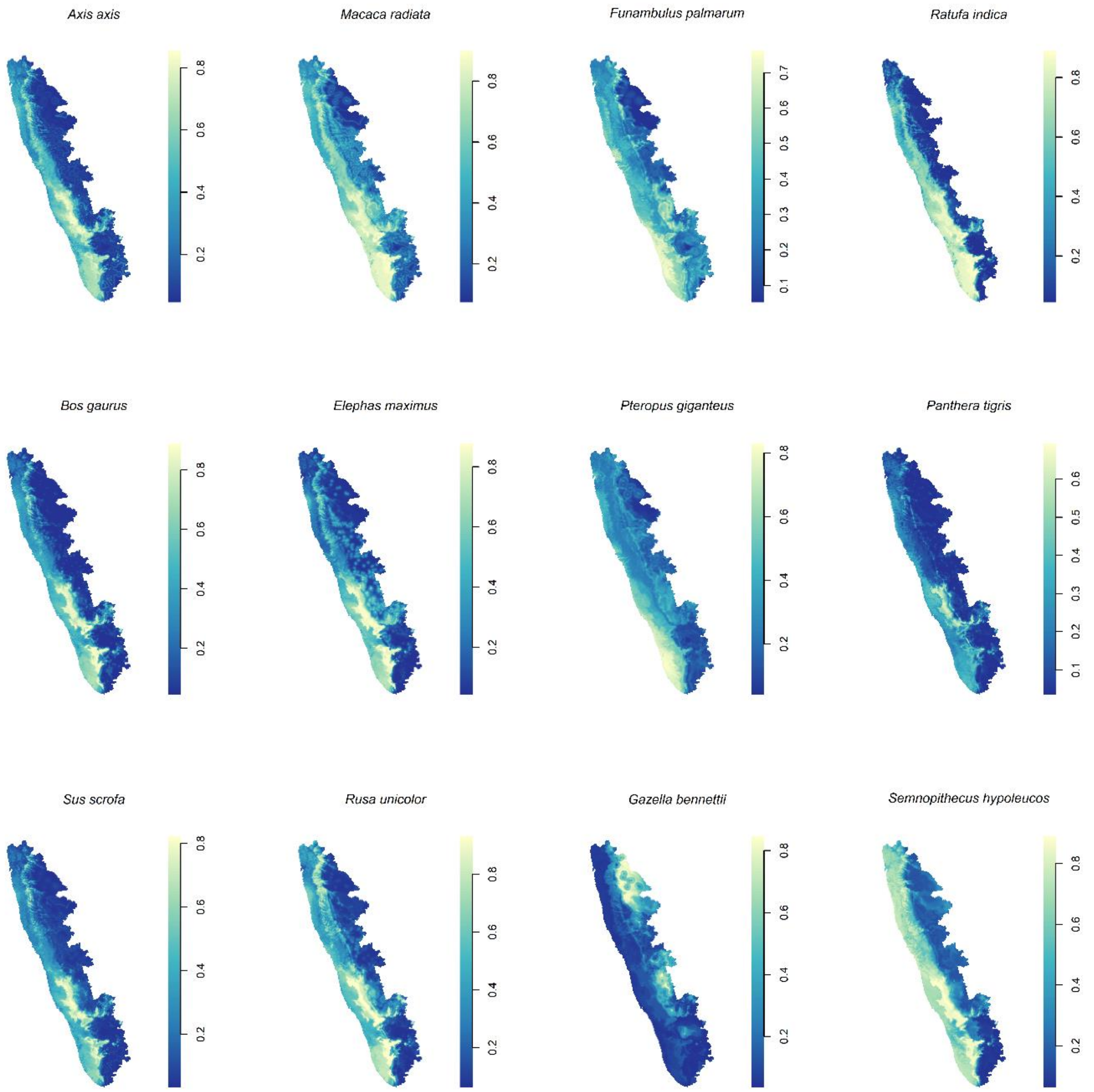

S3 Figure 2. Individual species distributions.

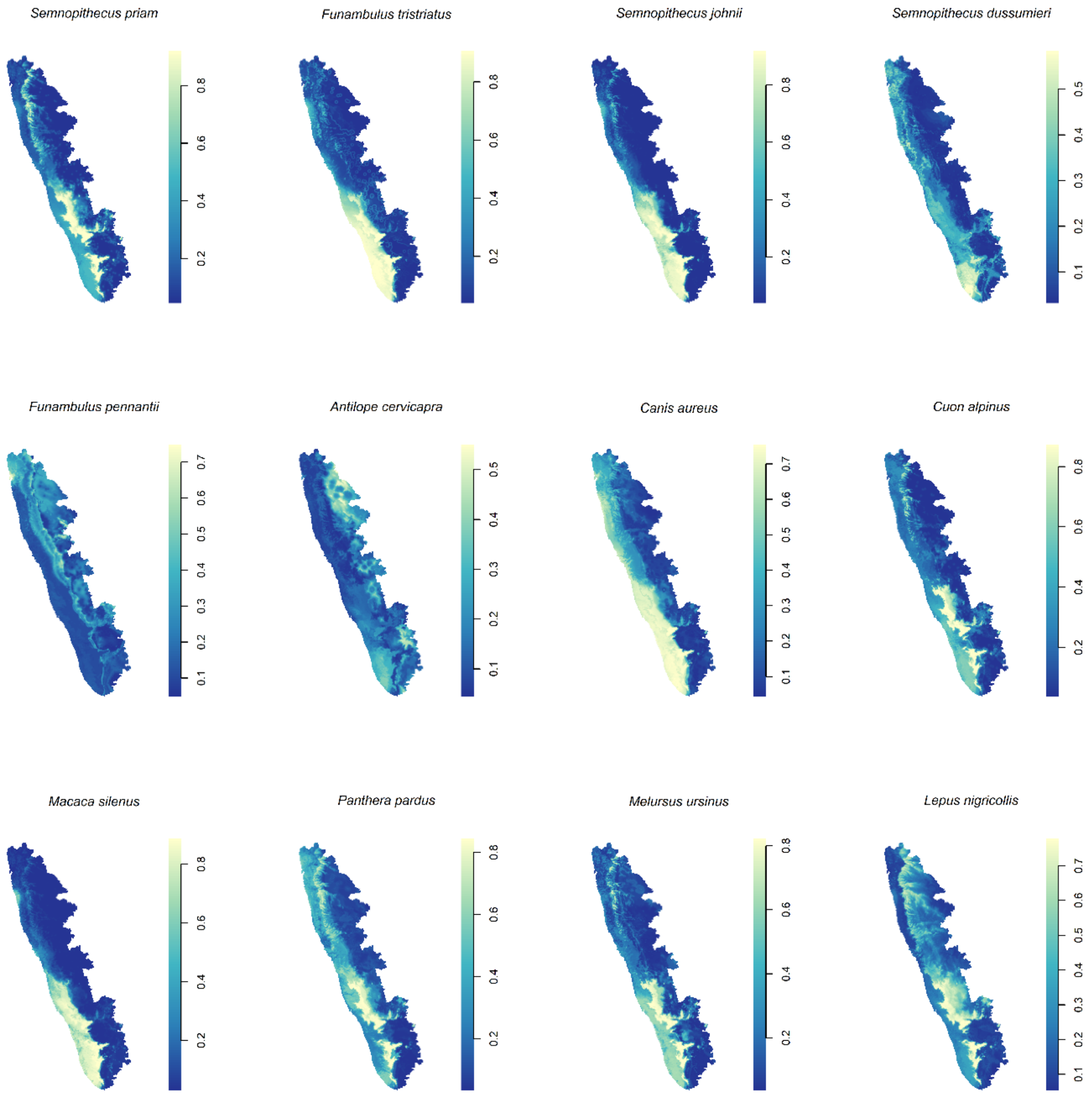

S4 Table 2. Secondary analysis species distribution ensemble models based on the weighted means of boosted regression tree-, random forest-, and generalised additive model-based species distributions. These models used an alternate India-wide sample of mammalian observations for a sensitivity analysis comparison of species richness estimates based on regional (S1 Table 1) and country-wide samples. Performance is indicated by the area under the receiver operating characteristic curve (AUC) and fit is indicated by the deviance. The true skill statistic was used to mark the cutoff of the estimated species distribution function demarcating species presence.

**Mammal species**	**Observations**	**AUC (%)**	**TSS**	**Deviance**
Axis axis	626	96	0.81	0.57
Macaca radiata	360	89	0.67	0.80
Funambulus palmarum	252	91	0.73	0.74
Ratufa indica	257	97	0.91	0.49
Bos gaurus	253	96	0.81	0.53
Elephas maximus	252	98	0.89	0.43
Pteropus giganteus	242	88	0.61	0.88
Panthera tigris	339	95	0.80	0.59
Sus scrofa	351	87	0.69	0.88
Rusa unicolor	297	93	0.74	0.71
Gazella bennettii	159	90	0.70	0.77
Semnopithecus hypoleucos	92	86	0.66	0.93
Semnopithecus priam	56	87	0.65	0.87
Funambulus tristriatus	50	96	0.90	0.51
Semnopithecus johnii	50	91	0.87	0.65
Semnopithecus dussumieri	330	91	0.70	0.74
Funambulus pennantii	431	82	0.58	0.99
Antilope cervicapra	171	89	0.80	0.74
Canis aureus	322	88	0.68	0.83
Cuon alpinus	61	91	0.70	0.77
Macaca silenus	34	81	0.61	0.87
Panthera pardus	108	82	0.57	1.03
Melursus ursinus	64	85	0.60	0.95
Lepus nigricollis	66	65	0.40	1.33
Boselaphus tragocamelus	398	93	0.80	0.64
Herpestes edwardsii	55	79	0.54	1.08
Paradoxurus hermaphroditus	48	64	0.44	1.24
Vulpes bengalensis	40	77	0.54	1.01
Hyaena hyaena	39	70	0.50	0.89
Lutrogale perspicillata	30	86	0.72	0.76

S5 Figure 3. Individual species distributions estimated with alternate country-wide sample.

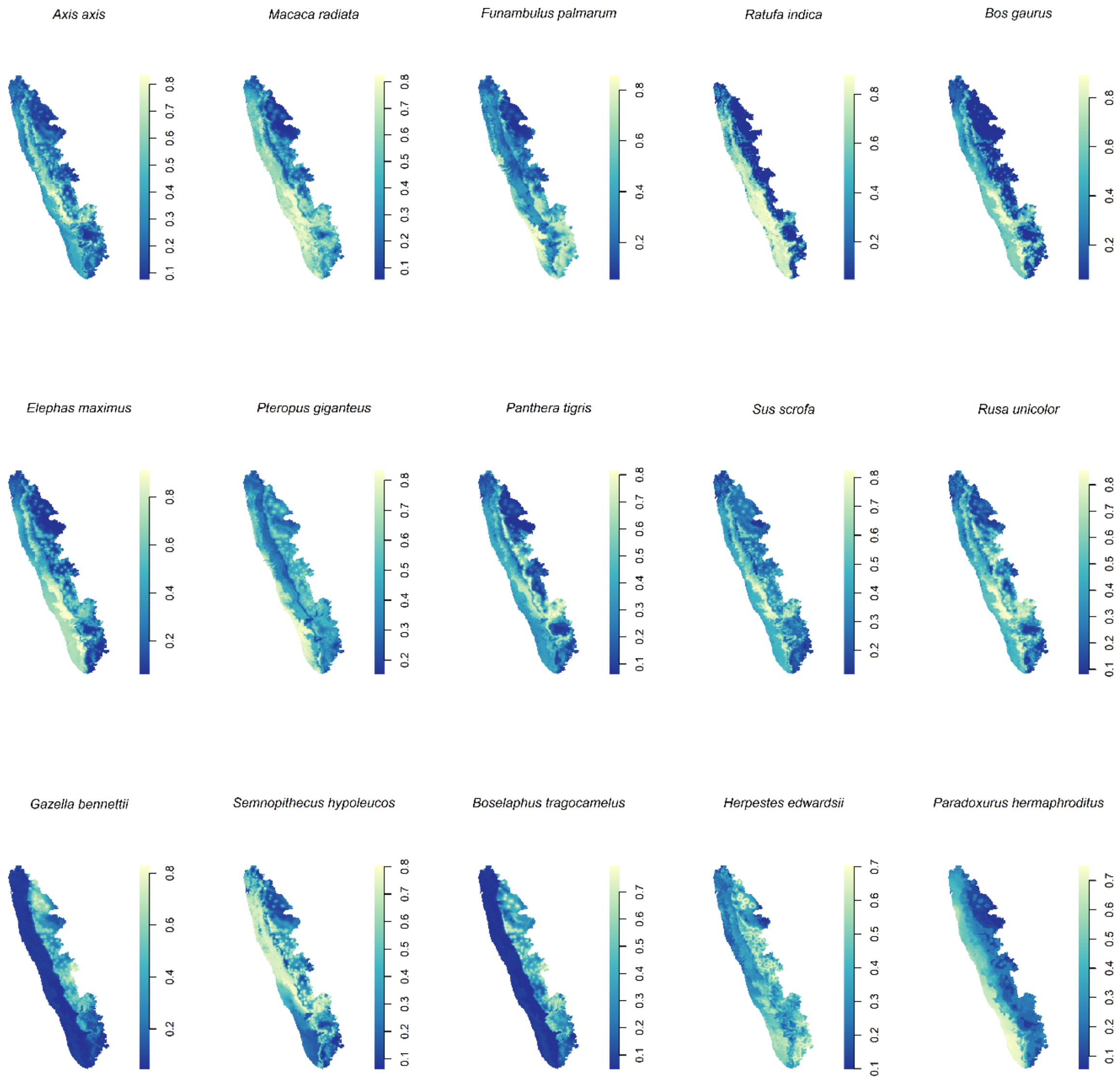

S6 Figure 4. Individual species distributions estimated with alternate country-wide sample.

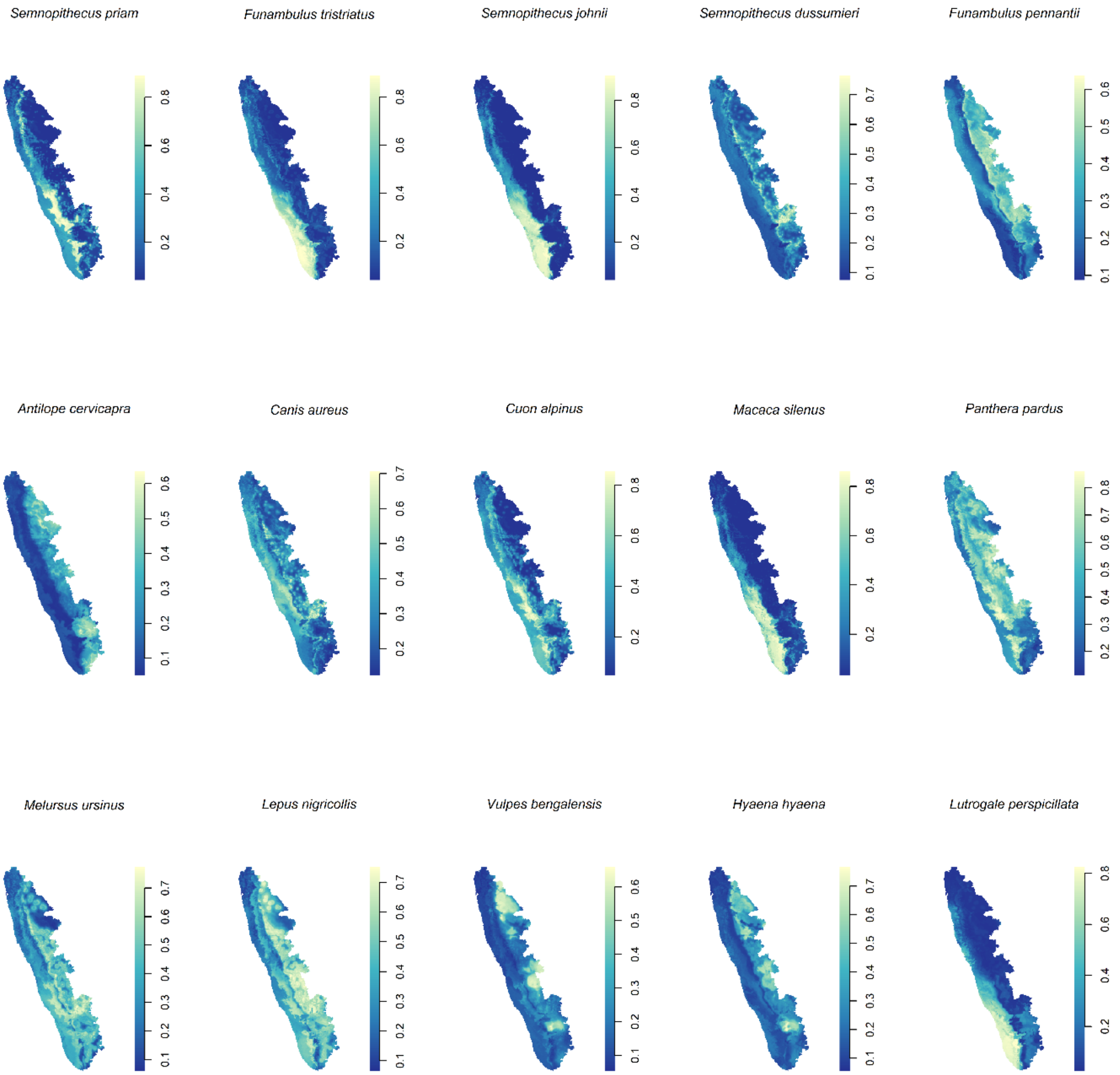

S7 Table 3. Regression coefficients and 95% confidence intervals for the associations between Kyasanur Forest disease virus outbreaks and landscape features as derived from inhomogeneous Poisson models of the outbreak point process and using the alternate country-wide sample to estimate species richness. Coefficients represent adjusted associations wherein each landscape feature is adjusted for all other features included in the multiple point process model. The Akaike information criterion (AIC) and area under the receiver operating characteristic curve (AUC) show model fit and performance, respectively.

**Landscape features**	**Coefficient**	**95% confidence interval**	**AIC**	**AUC**
Mammal richness	0.59	0.25 – 0.93	-5.09	0.71
Forest loss (deciles)	4.99	2.51 – 7.45		
Mammal richness:forest loss interaction	-0.35	-0.55 – -0.16		
Annual precipitation (100 mm)	0.04	0.01 – 0.06		
Annual temperature (Celsius)	-0.01	-0.02 – 0.001		

S8 Figure 5. The distribution of Kyasanur Forest disease virus outbreak risk derived from the inhomogeneous Poisson model, and using the alternate country-wide sample to estimate species richness, is presented in the centre panel with the lower and upper 95% confidence limits to the left and right, respectively.

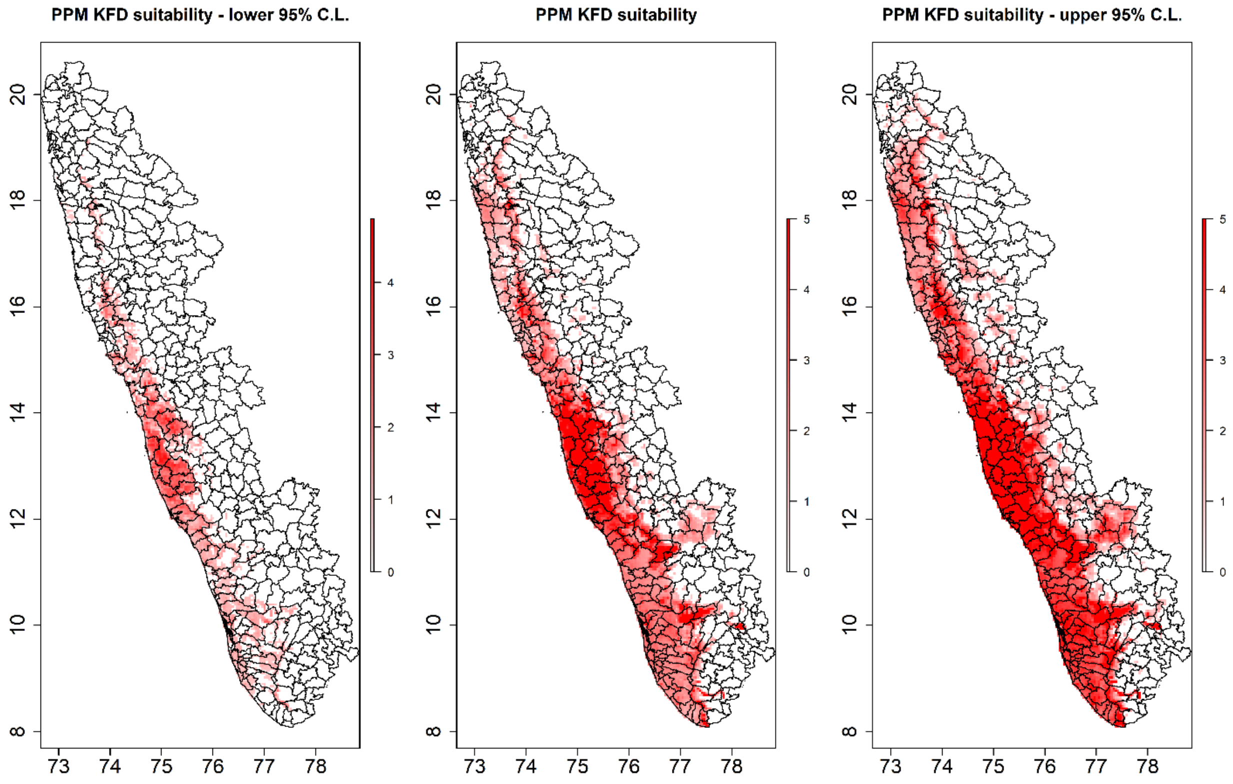

S9 Table 4. Taluk-level multiple integrated nested Laplace approximation model (zero-inflated Poisson family) of Kyasanur Forest disease virus outbreaks. Mammal species richness was adjusted for the biotic constraints of sympatric species using the SESAM framework. The Watanabe-Akaike information criterion (WAIC) was used to assess model fit.

**Landscape features**	**Coefficient**	**95% credible interval**	**WAIC**
Mammal richness$_{SESAM}$	1.51	0.40 – 2.63	215.16
Forest loss (deciles)	8.32	2.55 – 13.88	
Mammal richness:forest loss interaction	-0.80	-1.39 – -0.20	
Annual precipitation (100 mm)	0.10	0.01 – 0.20	
Annual temperature (Celsius)	0.003	-0.045 – 0.044	

S10 Figure 6. The distribution of Kyasanur Forest disease virus suitability derived from the integrated nested Laplace approximation model (zero-inflated Poisson family) is presented in the centre panel with the lower and upper 95% credible limits to the left and right, respectively.

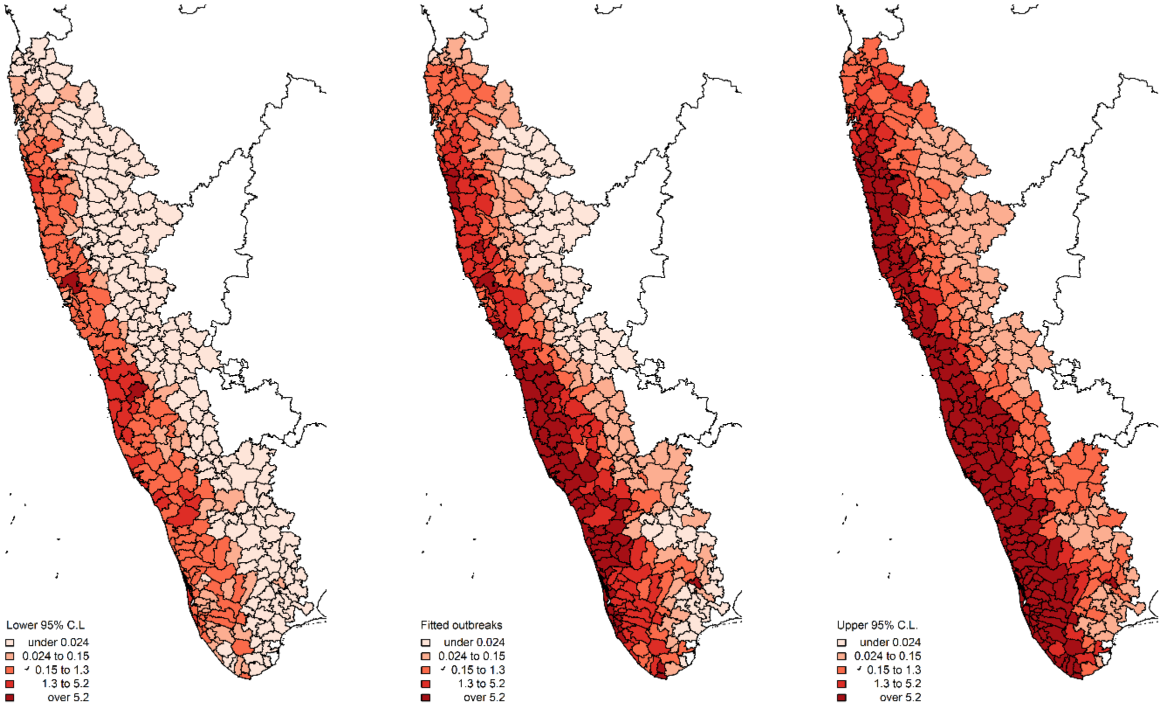

S11 Table 5. Taluk-level integrated nested Laplace approximation model (binomial family) of Kyasanur Forest disease virus outbreaks using the alternate country-wide sample to estimate species richness. Mammal species richness was adjusted for the biotic constraints of sympatric species using the SESAM framework. The Watanabe-Akaike information criterion (WAIC) was used to assess model fit.

**Landscape features**	**Coefficient**	**95% credible interval**	**WAIC**
Mammal richness$_{SESAM}$	1.25	0.48 – 2.08	151
Forest loss (deciles)	8.00	1.37 – 14.50	
Mammal richness:forest loss interaction	-0.75	-1.34 – -0.18	
Annual precipitation (100 mm)	0.00	0.00 – 0.1	
Annual temperature (Celsius)	-0.02	-0.038 – -0.001	

S12 Figure 7. The adjusted distribution of mammalian species richness among the Western Ghats using the alternate country-wide sample. Species richness was adjusted using the SESAM framework to introduce biotic constraints to adjust the presence of each species for the presence of other species within each taluk.

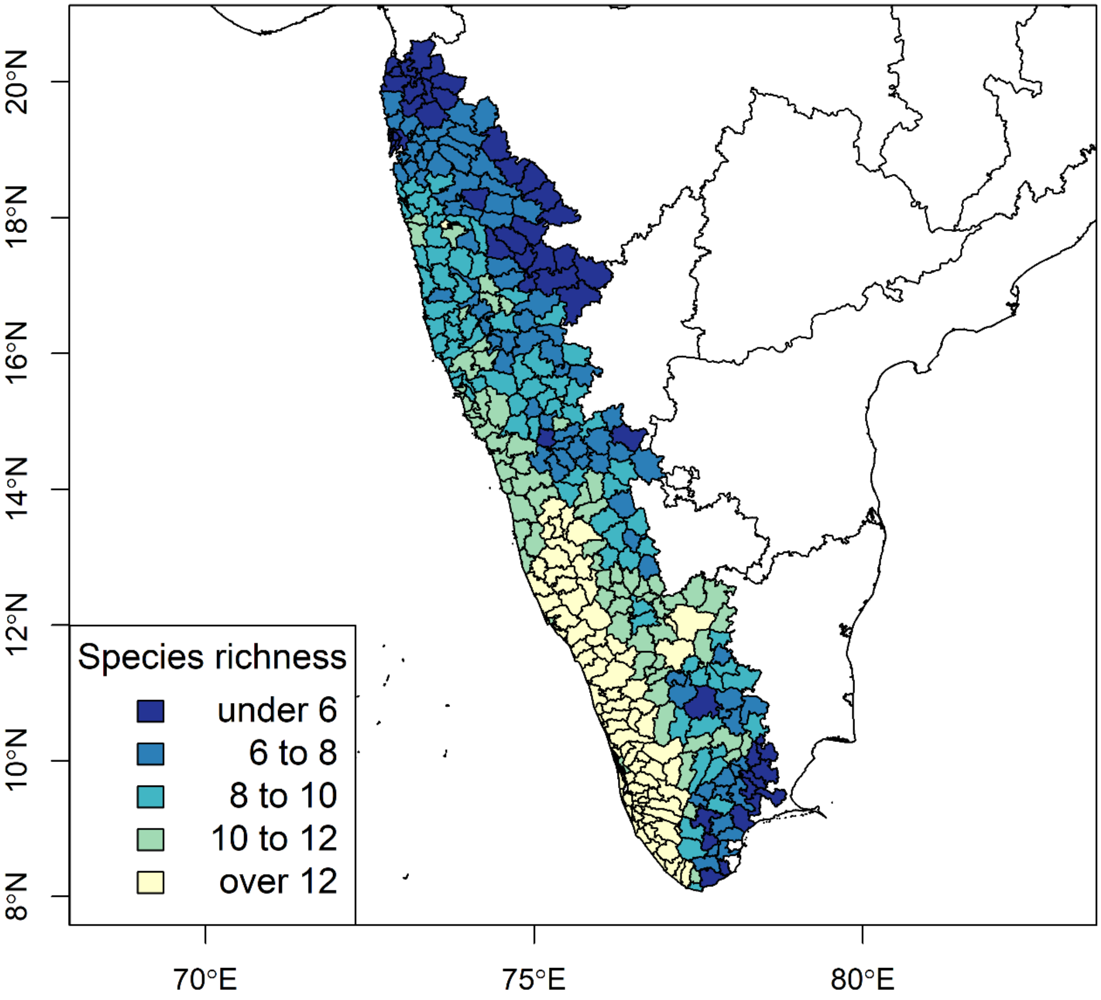

S13 Table 6. Unadjusted regression coefficients and 95% confidence intervals for the crude associations between Kyasanur Forest disease virus outbreaks and individual mammal species as derived from inhomogeneous Poisson models of the outbreak point process. The Akaike information criterion (AIC) and area under the receiver operating characteristic curve (AUC) show model fit and performance, respectively.

Mammal species	Coefficient	95% confidence interval	AIC	AUC
Axis axis	4.17	2.87 – 5.49	20.54	0.70
Macaca radiata	3.60	2.25 – 4.96	32.82	0.65
Funambulus palmarum	3.63	2.03 – 5.22	43.92	0.61
Ratufa indica	2.64	1.52 – 3.76	13.07	0.62
Bos gaurus	3.02	1.95 – 4.09	33.89	0.67
Elephas maximus	2.59	1.51 – 3.66	42.69	0.61
Pteropus giganteus	0.44	-1.01 – 1.89	64.14	0.53
Panthera tigris	1.76	-0.06 – 3.59	61.15	0.63
Sus scrofa	3.31	2.14 – 4.48	36.06	0.67
Rusa unicolor	2.84	1.74 – 3.94	37.57	0.64
Gazella bennettii	-4.97	-8.03 – -1.90	46.85	0.58
Semnopithecus hypoleucos	5.54	3.68 – 7.41	4.73	0.70
Semnopithecus priam	2.57	1.66 – 3.48	37.19	0.65
Funambulus tristriatus	0.18	-0.76 – 1.12	64.34	0.59
Semnopithecus johnii	0.76	-0.10 – 1.62	61.67	0.67
Semnopithecus dussumieri	2.52	0.72 – 4.32	57.46	0.74
Funambulus pennantii	-9.71	-14.74 – -4.68	42.00	0.62
Antilope cervicapra	-11.68	-17.13 – -6.22	35.94	0.50
Canis aureus	2.41	1.29 – 3.53	46.12	0.67
Cuon alpinus	1.63	0.58 – 2.69	56.28	0.65
Macaca silenus	0.45	-0.50 – 1.39	63.65	0.67
Panthera pardus	3.52	2.38 – 4.66	27.90	0.67
Melursus ursinus	1.14	0.003 – 2.28	60.89	0.65
Lepus nigricollis	2.73	1.49 – 3.98	47.72	0.56

S14 Table 7. Taluk-level regression coefficients and 95% credible intervals for the associations between Kyasanur Forest disease virus outbreaks and each species abundance (per 10 species-present pixels per taluk) and relative abundance as derived from integrated nested Laplace approximation models (binomial family) of Kyasanur Forest Disease virus outbreaks. Coefficients represent crude associations from bivariate models.

**Mammal species abundance/relative abundance**	**Coefficient**	**95% credible interval**	**WAIC**
Axis axis abundance	0.02	0.00 – 0.04	162.74
Axis axis relative abundance	***29.09***	***3.84 – 51.45***	***159.07***
Macaca radiata abundance	***0.01***	***0.015 – 0.02***	***155.13***
Macaca radiata relative abundance	-0.035	-2.85 – 2.20	164.55
Funambulus palmarum abundance	0.01	-0.02 – 0.03	165.39
Funambulus palmarum relative abundance	-1.17	-6.37 – 2.61	164.39
Ratufa indica abundance	-0.01	-0.10 – 0.05	164.82
Ratufa indica relative abundance	-10.08	-60.53 – 32.34	163.04
Bos gaurus abundance	0.01	-0.01 – 0.02	164.05
Bos gaurus relative abundance	3.84	-10.99 – 15.75	164.12
Elephas maximus abundance	0.03	-0.03 – 0.07	163.88
Elephas maximus relative abundance	12.16	-37.16 – 55.51	163.19
Pteropus giganteus abundance	-0.02	-0.09 – 0.02	163.96
Pteropus giganteus relative abundance	-17.76	-47.49 – 3.83	160.01
Panthera tigris abundance	0.00	-62.09 – 62.03	162.69
Panthera tigris relative abundance	0.00	-62.09 – 62.03	162.69
Sus scrofa abundance	0.01	-0.01 – 0.02	163.80
Sus scrofa relative abundance	12.22	-7.62 – 28.79	162.97
Rusa unicolor abundance	0.01	0.00 – 0.02	162.88
Rusa unicolor relative abundance	-0.20	-6.44 – 4.39	164.90
Gazella bennettii abundance	-0.01	-0.03 – 0.01	178.54
Gazella bennettii relative abundance	-10.34	-53.79 – 18.15	175.72
Semnopithecus hypoleucos abundance	***0.02***	***0.01 – 0.03***	***139.72***
Semnopithecus hypoleucos relative abundance	***2.24***	***0.98 – 3.53***	***152.75***
Semnopithecus priam abundance	***0.01***	***0.001 – 0.02***	***160.23***
Semnopithecus priam relative abundance	5.64	-0.40 – 11.31	161.87
Funambulus tristriatus abundance	-0.01	-0.06 – 0.02	164.13
Funambulus tristriatus relative abundance	-17.00	-49.30 – 7.78	161.69
Semnopithecus johnii abundance	0.00	-0.01 – 0.01	164.51
Semnopithecus johnii relative abundance	-6.22	-22.30 – 6.67	163.55
Semnopithecus dussumieri abundance	-3.01	-40.37 – 19.42	163.68
Semnopithecus dussumieri relative abundance	-3.01	-40.37 – 19.42	163.68
Funambulus pennantii abundance	-0.82	-28.21 – 14.46	170.30
Funambulus pennantii relative abundance	-9.05	-55.73 – 22.27	163.78
Antilope cervicapra abundance	-0.02	-0.08 – 0.03	179.59
Antilope cervicapra relative abundance	-13.24	-63.62 – 22.42	166.38
Canis aureus abundance	0.00	-0.02 – 0.01	166.73
Canis aureus relative abundance	-4.86	-12.21 – 1.00	162.24
Cuon alpinus abundance	0.00	-0.02 – 0.02	164.38
Cuon alpinus relative abundance	6.17	-20.42 – 27.85	164.18
Macaca Silenus abundance	0.00	-0.01 – 0.01	165.03
Macaca Silenus relative abundance	-5.17	-15.02 – 2.76	163.12
Panthera pardus abundance	***0.02***	***0.01 – 0.03***	***147.76***
Panthera pardus relative abundance	***5.06***	***1.79 – 8.25***	***156.37***
Melursus ursinus abundance	0.01	-0.01 – 0.02	163.44
Melursus ursinus relative abundance	11.54	-4.69 – 25.33	162.64
Lepus nigricollis abundance	0.01	0.00 – 0.02	162.10
Lepus nigricollis relative abundance	2.26	-8.24 – 10.70	164.28

S15 Figure 8. Taluk-level species abundance and relative abundance for primate species associated with KFDV outbreaks (S14 Table 7).

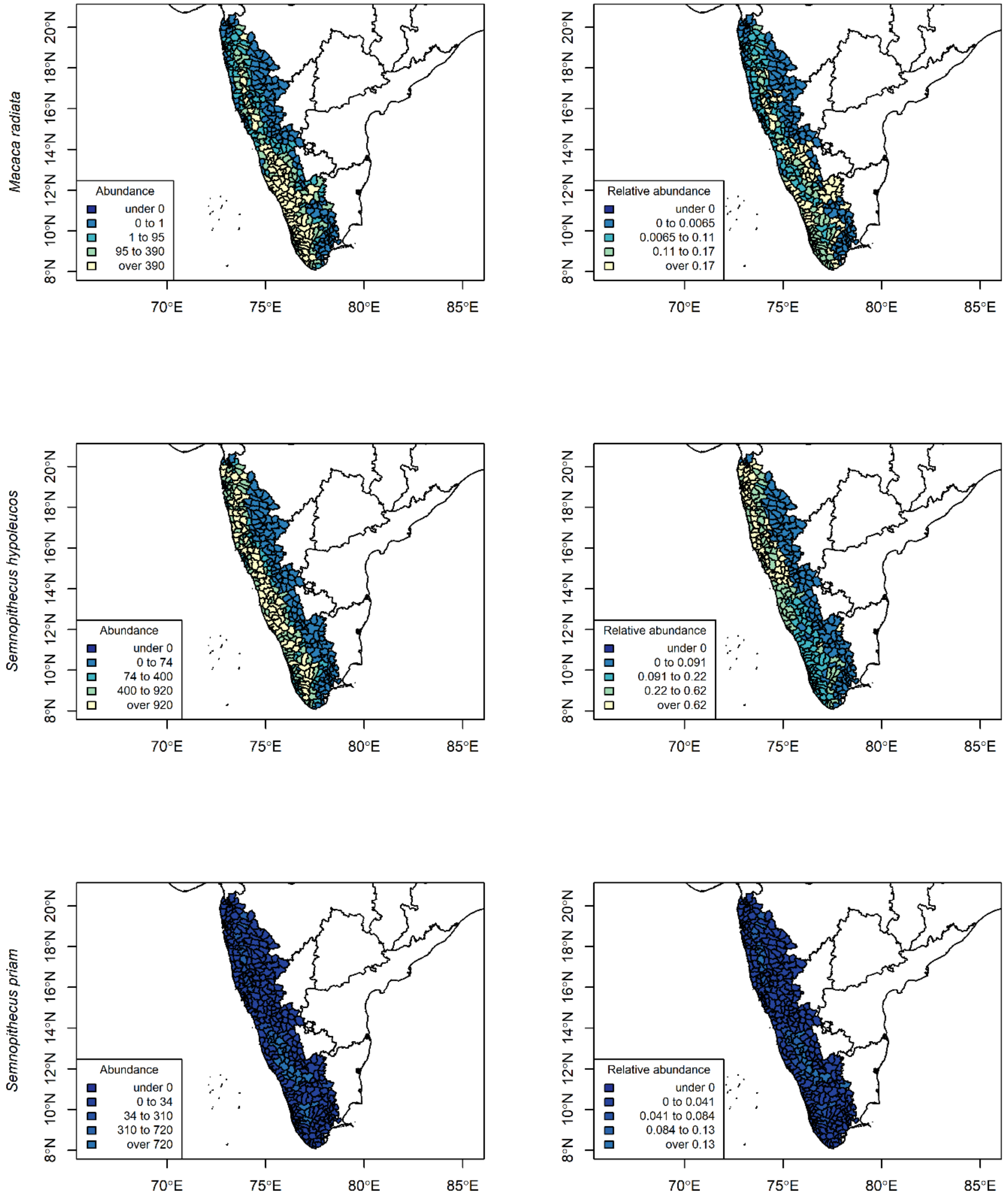

Supplement: Supplementary file 1 — Supplementary material [file mmc1.pdf]
